# Current Evidence of the Application of Music in Tai Chi Exercise: Scoping Review

**DOI:** 10.2196/60104

**Published:** 2024-09-19

**Authors:** Yan Du, Gao-Xia Wei, Yichao He, Hongting Ning, Penny Roberts, Edward Golob, Zenong Yin

**Affiliations:** 1 School of Nursing, University of Texas Health Science Center at San Antonio San Antonio, TX United States; 2 Department of Psychology University of Chinese Academy of Sciences Beijing China; 3 Xiangya School of Nursing Central South University Changsha China; 4 School of Music Loyola University New Orleans New Orleans, LA United States; 5 Department of Psychology University of Texas at San Antonio San Antonio, TX United States; 6 Department of Public Health University of Texas at San Antonio San Antonio, TX United States

**Keywords:** Tai Chi, exercise, music, synergetic effects, review, scoping review, thematic analysis, health outcome, motivation, performance, dissemination, implementation, public health, data extraction

## Abstract

**Background:**

Music has frequently been used in movement exercises to enhance health benefits. However, scientific evidence regarding the application of music to Tai Chi practice is limited.

**Objective:**

This scoping review aims to understand how music has been used in Tai Chi practice and whether music could be applied to Tai Chi practice to help optimize its benefits.

**Methods:**

PubMed, CINAHL, CNKI, and Weipu databases were searched. We included studies that compare Tai Chi practice experience or health outcomes between individuals practicing Tai Chi with music and those practicing Tai Chi without music. Studies published through September 2022 were identified. Two researchers (YD and YH) independently performed study selection and data extraction. Thematic analysis was used to summarize and categorize the findings of the included studies.

**Results:**

Seven studies were included in this review. All 7 included studies are experimental studies. Practicing Tai Chi with music might lead to positive perceptions of Tai Chi practice (eg, motivation, concentration, enjoyment, compliance, and performance) and higher evaluations of Tai Chi instructional quality, especially for Tai Chi beginners. The effects of incorporating music into Tai Chi practice on health outcomes are inconclusive due to the heterogeneities of the sample size, and the intervention components, lengths, and frequencies of the included studies.

**Conclusions:**

Applying music to Tai Chi practice may result in positive Tai Chi practice experience and adherence, particularly for beginners, which could help improve the dissemination and implementation of Tai Chi interventions for public health. However, whether applying music to Tai Chi practice leads to synergetic effects on health outcomes needs further investigation.

## Introduction

Tai Chi, originating in China, is an ancient Chinese martial art that evolved into a sport and mind-body exercise. It is characterized by slow and gentle movements, deep breathing, and meditation. There are various types of Tai Chi such as Chen, Yang, Hao, Wu, and Sun styles [1]. Among these, Chen style is the oldest, characterized by slow and smooth movements with fast and explosive ones; Yang Style is the most widely practiced globally, characterized by slow, gentle, and flowing movements [1]. Each style may include simplified forms, traditional forms, short forms, and long forms. A Tai Chi form is a sequence of movements that are performed in a slow, continuous, and flowing manner, integrating principles of balance, relaxation, and alignment [1,2].

Widely recognized as a valuable therapeutic intervention, Tai Chi is often recommended by health professionals and embraced by the public to complement conventional medical treatments [3]. Its slow and deliberate movements, encompassing aerobic, stretching, balance, and strengthening exercises, have garnered increasing attention among populations with various health statuses and within the scientific community for their potential health benefits [4-6]. Evidence-based research underscores Tai Chi’s health benefits, including but not limited to improvements in balance [7,8], muscle strength [9], cardiovascular and metabolic health [10-13], physical and cognitive function [14-18], mental health [19,20], and quality of life [21-24].

Incorporating music into movement exercises is a common practice to enhance ergogenic effects [25]. In China, Tai Chi practitioners often use soft, relaxing Chinese folk music to promote relaxation and concentration during Tai Chi practice [26,27]. Using music during Tai Chi practice has also been reported in populations outside of China [28,29]. While studies examining Tai Chi’s effects on health outcomes are on the rise, few have explored the use of music during Tai Chi practice in Western populations [30-32]. Previous research has inadequately addressed the application of music in Tai Chi practice [33], and studies assessing Tai Chi with music seldom investigate potential synergistic effects on health outcomes [34,35]. Furthermore, the ongoing debate surrounding the use of music during Tai Chi practice reflects different perspectives on music’s impact [36]. Some argue that Tai Chi is a form of moving meditation, suggesting that engaging in Tai Chi with music may hinder the attainment of “rujing” (the absence of idle thoughts or the cultivation of stillness). Conversely, others indicate that music can aid in reducing distractions and facilitating “rujing” by calming the mind [36]. Scientific evidence is thus imperative to guide best practices in incorporating music into Tai Chi for optimal health benefits.

Therefore, this study aims to explore current evidence regarding the application of music in Tai Chi practice through a scoping review of studies comparing Tai Chi practiced with music to that practiced without. Our objectives include understanding how music has been applied to Tai Chi practice, evaluating what specific outcomes were assessed in the current scientific literature, and exploring whether practicing Tai Chi with music offers advantages over practicing Tai Chi without music.

## Methods

### Study Design

We used the updated Arksey and O’Malley five-stage framework [37] to guide the conduct of the scoping review. Specifically, we followed these steps: (1) identifying the research question based on the research team’s experience in conducting Tai Chi–related research and reviewing current literature; (2) identifying relevant studies; (3) selecting the studies; (4) charting the data; and (5) collating, summarizing, and reporting results. Steps 2-5 are described in detail below. Additionally, we used the PRISMA-ScR (Preferred Reporting Items for Systematic Reviews and Meta-analysis extension for Scoping Reviews) guidelines [38] to draft and report this scoping review. The PRISMA-ScR checklist can be found in Multimedia Appendix 1. We registered this review at the Open Science Framework [39].

### Data Search

We searched the electronic databases PubMed and CINAHL in September 2022. Medical Subject Headings terms “Tai Chi” and “music” were used to build the search strategy on PubMed. The search strategies for PubMed are illustrated in Multimedia Appendix 2. Subsequently, the search terms used on CINAHL were constructed based on the PubMed search strategy. Given that Tai Chi originated in China, where the use of music during Tai Chi is common, we also searched Chinese databases CNKI and Weipu. “太极” (Tai Chi) and “音乐” (music) were used to search through Chinese databases. We did not set language or publishing year restrictions in the database searches as we aimed to comprehensively cover this topic in this scoping review.

### Eligibility Criteria

Inclusion criteria were (1) studies with comparisons between Tai Chi with music and Tai Chi without music, (2) published journal studies, and (3) at least 1 study group examining Tai Chi with music and 1 group examining Tai Chi without music. Given the broad scope of this scoping review, no age limit was set. Outcomes could encompass any measurements evaluated in the studies such as Tai Chi practice adherence and health outcomes. Exclusion criteria were (1) studies with nonempirical design, such as view paper, review paper, and protocols, and (2) experimental studies without comparisons between Tai Chi with and without music.

### Study Selection

First, all search records were merged, and duplicates were removed. The eligibility criteria were then discussed and refined by the study team based on the search processes. YD screened the title and abstract based on the inclusion and exclusion criteria. YD and YH independently screened the full papers according to the eligibility criteria. Any discrepancies were resolved through discussions and consultations with a third researcher (GXW).

### Data Extraction

YD drafted the data extraction form. YD and YH tested the extraction by charting 2 studies independently and refined the data extraction form accordingly. The form included study location, study design, sample characteristics, Tai Chi selection, music selection, assessed outcomes, and study findings. YD and YH independently extracted data from each study according to the extraction form. Any discrepancies were resolved through discussions and consultations with a third researcher (GXW). Data were extracted in the original published language, and a bilingual researcher translated the Chinese data into English after synthesis, which was then checked by a native English speaker.

### Synthesis

We used a narrative approach to summarize, analyze, and assess the evidence in this review. Specifically, one table charted the characteristics of included studies, and another table summarized the use of Tai Chi and music in each study. A thematic analysis approach [40] was adopted to summarize and categorize the findings of included studies. First, YD created a codebook based on the coding of the extracted data and summarized the findings for each code. Second, YH and GXW reviewed the codes and summary findings against the extracted data. Summary quotes are quotes summarized by the authors but not direct quotes from the studies included. Subsequently, YD, YH, and GXW categorized the codes into subthemes. The study team reviewed the themes, subthemes, codes, and summary quotes. Given the scoping nature of this review, no assessment of study quality was conducted.

## Results

**Figure 1:**
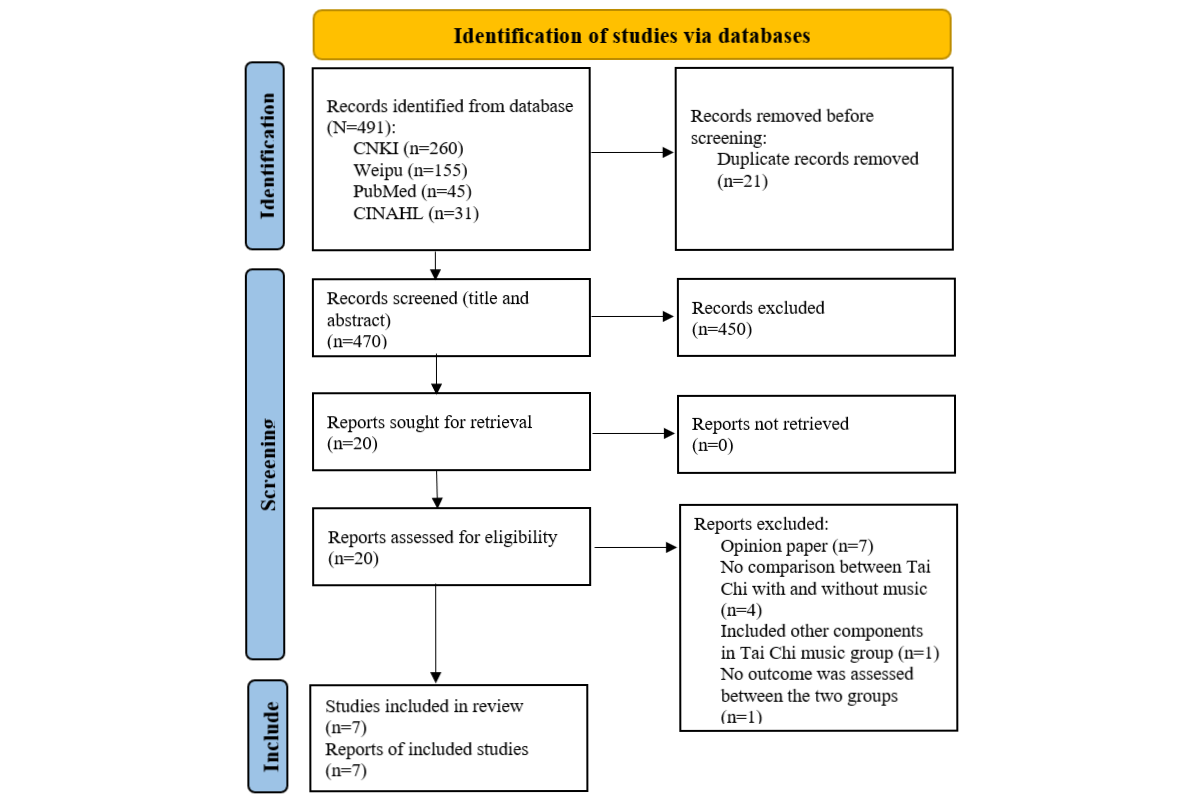
PRISMA diagram of selected studies. PRISMA: Preferred Reporting Items for Systematic Reviews and Meta-analysis.

A total of 491 papers in English or Chinese were identified, including 260 papers in CNKI, 155 papers in Weipu, 45 papers in PubMed, and 31 papers in CINAHL (Figure 1). We removed 21 duplicated papers, leaving 471 papers for the title and abstract screening. Of those, we considered 20 studies for full-text screening. We further excluded 13 papers after a full-text screening due to reasons of being opinion papers (n=7), lacking a comparison between Tai Chi with and without music (n=5), and involving other components in the Tai Chi with music group (n=1). Figure 1 indicates the reasons and numbers of excluded studies in each screening stage.

All 7 included studies are clinical trials (Table 1); 6 studies were conducted in China and 1 in the United States. Five of the 6 studies in China included only college students, and the rest focused on nurses with impaired mental health. The study conducted in the United States primarily included older adults. Five studies reported gender distributions; 2 reported 100% female, 1 reported 9% female, and the other 2 consisted of 53% and 47% female, respectively. All 7 studies included at least 2 study groups; 1 group for Tai Chi with music and another for Tai Chi without music. For studies with available intervention frequencies, it ranged from 14 to 16 sessions within 7 weeks (about 1 and a half months) to 3 months. The retention rate ranged from 72% to 100%. The sample size included in the final analysis ranged from 13 to 162 participants.

**Table 1:** Study characteristics.

Study	Location	Design	Population or setting	Age and sample size	Intervention	Frequency and length	Measurements	Retention
Du et al (2017) [31]	United States	Cluster randomized controlled trial	Adults in a senior day activity center	Age: 50-82 years old Intervention (n=12): 72.3, SD 5.7 Control (n=6): 62.0, SD 10.3 Gender: 95.5% female; 100% female retained	Intervention: Tai Chi with music Control: Tai Chi without music	Once a week for 15 weeks. Recorded video was sent home to promote home practice	Dynamic gait index Fear of falling Tai Chi practice compliance	72%
Li (2013) [41]	China	Randomized controlled trial	Students at a local university	Age: not available. Intervention (n=30). Control (n=29). Gender: 100% female	Intervention: Tai Chi with music Control: Tai Chi without music	Not mentioned	Tai Chi learning motivation Concentration Satisfaction with instruction Overall performance (correctness, consistency, pace, style, overall)	100%
Liu et al (2020) [19]	China	Randomized controlled trial	Registered nurses with impaired mental health working in the operation room in 4 local public tertiary hospitals	Age: not available Intervention: Tai Chi with music (n=30); Tai Chi only (n=30); music only (n=30). Control: nonactive (n=30). Gender: not available	Intervention (three groups): (1) Tai Chi with music; (2) Tai Chi only; (3) music only. Control: nonactive	August 2018 to October 2018	Physical symptom, depression, and anxiety indicators (SCL-90^a^) Coping style (SCSQ^b^)	100%
Lv et al (2019) [42]	China	Cluster controlled trial	Students at a local university	Age: not available. Tai Chi beginner (n=16). Tai Chi proficient (n=16). Gender: not available	Tai Chi beginner (four scenarios): (1) no music; (2) relaxing and soft music; (3) joyful and fast music; (4) sad music Tai Chi proficient (four scenarios): (1) no music; (2) relaxing and soft music; (3) joyful and fast music; (4) sad music	Not mentioned	Interest in Tai Chi (PESIS^c^) Galvanic skin response	100%
Zhang (1999) [43]	China	Randomized controlled trial	Students at a local university	Age: not available Intervention (n=76) Control (n=86) Gender: 47% female	Intervention: Tai Chi with music Control: Tai Chi without music	From September 1997 to July 1998	Tai Chi performance evaluated by Tai Chi instructors	100%
Zhang (2009) [44]	China	Randomized controlled trial	Students at a local medical school	Age: not available Intervention (n=70) Control (n=74) Gender: 53% female	Intervention: Tai Chi and Tai Chi sword with music Control: Tai Chi and Tai Chi sword without music	Twice a week for 7 weeks.	Tai Chi performance evaluated by two Tai Chi instructors Practice outside of class	100%
Zhao and Wang (2017) [45]	China	Randomized controlled trial	Students at a vocational and technical college	Age: not available Intervention (n=49) Control (n=44) Gender: 9% female	Intervention: Tai Chi with music Control: Tai Chi without music	16 sessions from September 2016 to December 2016	Interests in Tai Chi Perceived difficulties of learning Tai Chi Positive emotion inspiration Instruction quality Tai Chi performance evaluated by two Tai Chi instructors	100%

^a^SCL-90: Symptom Checklist-90.

^b^SCSQ: Simplified Coping Style Questionnaire.

^c^PESIS: Sports Situational Interest Scale Chinese version.

Table 2 shows the Tai Chi styles or forms, the music type being used, and how the study applied music to Tai Chi practice. Three of the 7 studies specified Tai Chi styles and forms (the 24-form simplified Yang style). There were variations in music selections used for Tai Chi practice. In the study conducted in the United States, a board-certified music therapist selected the music; others did not specify how the music was selected or by whom. Various music types were selected, but the studies in China used Chinese music or Chinese medicine principle-oriented music; while the US study used Western music with instruments familiar to the ethnic groups. Five of the 7 studies described how they used music during Tai Chi practice. Among the 5 studies, 3 studies did not use music during Tai Chi instructions and used music only when practicing Tai Chi routines; 1 study used music throughout the Tai Chi sessions. The remaining study did not provide Tai Chi instructions; however, it assessed body responses to diverse types of music while practicing Tai Chi routines.

Thematic analysis revealed two major themes with corresponding subthemes: health outcomes including (1) physical health and (2) mental and emotional health; and experience of Tai Chi practice including (1) Tai Chi practice and (2) perceived instruction quality (Table 3). Included studies assessed various outcomes to compare the efficacy of Tai Chi with and without music. First, the assessed health outcomes included but were not limited to physical and mental health. Overall, practicing Tai Chi with music led to significantly better balance (dynamic gait index); and a trend of better physical health indicators and better mental health (depression and coping), which were not significant. Tai Chi beginners had significantly higher skin response to Tai Chi with slow, gentle music or relaxing, joyful music than advanced practitioners; higher skin response was found in advanced practitioners compared to those beginners when practicing Tai Chi without music. Second, for the experience of Tai Chi practice, at least 1 study showed that practicing Tai Chi with music led to multiple outcomes such as higher adherence in and outside of class [31,44], more confidence in learning and mastering Tai Chi [44,45], better concentration and enjoyment [41], less perceived difficulties [45], and better performance [41,43-45]. Third, instruction quality was assessed in 3 studies, 2 of which reported a higher satisfaction rate in the Tai Chi with music groups [41,44], and more participants rated the instruction high quality in the Tai Chi with music group compared to the control group [43].

**Table 2:** Tai Chi and music selection.

Study	Tai Chi selection	Music selection	The use of music for Tai Chi practice
Du et al (2017) [31]	The first 12 forms of the 24-form simplified Yang-style Tai Chi	Music was selected by a board-certified music therapist who had worked with older adults for over 10 years. For this study, Western music was selected to provide a grounded atmosphere—familiar, comfortable, and easily integrated into the background—rather than Eastern music, whose different tonal structure, harmony, or rhythmic structure may have been a distraction. The music selected aimed to provide a rhythmic structure intended to guide but not fully direct or entrain movement. Harmony was similarly considered, with the musical selection using Western 12-tone major and minor scales. The instrumentation of the selected music was Western (nature sounds, flute, using instruments familiar to the ethnic group largely represented by our sample).	The same recorded asynchronous instrumental music with nature sounds was used at each session for TC + M^a^ class, when participants performed learned Tai Chi forms. The music therapist was present in each class for assistance and consultation. All new Tai Chi forms were instructed in silence; asynchronous music, which means there is no conscious attempt from the individual to match their movements with the rhythm of the music, was only used while practicing previously learned movements.
Li (2013) [41]	The 1-12 forms of the 24-form simplified Yang-style Tai Chi	Not mentioned.	First, the instructor demonstrated performing two forms with music, which were instructed later. Second, instructing the two forms without music. Third, participants practiced Tai Chi with music following the instructor. Fourth, participants practiced Tai Chi with music. Fifth, the instructor corrected postures and movements without music, and finally, participants practiced Tai Chi with music.
Liu et al (2020) [19]	The 24-form simplified Yang-style Tai Chi	The principle of five-element music therapy was adopted. It is believed that ancient Chinese music consists of 5 notes (gong, shang, jiao, zhi, and yu), which were collected with the 5 elements of nature (metal, wood, water, fire, and earth). In addition, the 5 elements correspond to the 5 main body organs (heart, liver, spleen, lungs, and kidneys). Music was selected based on participants’ health status per Chinese medicine principles.	Not mentioned.
Lv et al (2019) [42]	Tai Chi (style not mentioned)	Relaxing and soft music, joyful and fast music, and sad music, respectively.	Each of the three types of music was randomly selected and applied to Tai Chi practice. The skin response test was done after participants felt calm and continued feeling calm even after the test was initiated in 30 seconds. Then participants started practicing Tai Chi with each type of music. PESIS^b^ was completed right after practicing with each type of music and practicing with each type of music was done for at least 5 minutes.
Zhang (1999) [43]	Tai Chi and Tai Chi sword	Different and appropriate music was selected for Tai Chi and Tai Chi sword accordingly.	Not mentioned.
Zhang (2009) [44]	Tai Chi (style not mentioned)	Fishermen’s Song at Eventide, which is characterized by meaningfulness and soothe, aligning with the feature of Tai Chi, was used.	Music was played while the instructor was demonstrating the whole Tai Chi routine or when participants were practicing learned forms.
Zhao and Wang (2017) [45]	Tai Chi (style not mentioned)	Music was selected to align the Tai Chi style with the rhythm and pace of music, as well as the demographics and cultural background of Tai Chi practitioners. The music being used was popular Chinese music.	First, one music was used during warm-up exercise, followed by another music, which is enthusiastic, to increase the willingness to learn of students; Second, the combination of joyful, relaxing, and victory music was played while the instructor provided the opening of the class. Third, the instructor demoed Tai Chi three times with music and then instructed each form with music in the background.

^a^TC + M: Tai Chi and music.

^b^PESIS: Sports Situational Interest Scale Chinese version.

**Table 3:** Themes of outcomes, and study findings between those who practiced Tai Chi with music and those without music.

Themes and findings					Author
**Health outcomes**					
	**Physical health**				
		DGI^a^	The post-DGI score in Tai Chi with music group was significantly higher than Tai Chi without music group after adjusting age.	Du et al (2017) [31]	
		Physical symptoms	There was a trend of better physical health indicators in Tai Chi with music group than Tai Chi only group or music only group. However, this was not significant.	Liu et al (2020) [19]	
		Galvanic skin response	Tai Chi beginners had significantly higher skin response values compared to advanced practitioners when practicing Tai Chi with all 3 types of music (more obvious effects to less: soft and relaxing, fast and joyful, and sad music) compared to more advanced users. However, in the Tai Chi group without music, there were no differences in skin response between beginners and the more advanced. Listening to sad music had the lowest response in both beginner and advanced groups.	Lv et al (2019) [42]	
	**Mental and emotional health**				
		Depression, anxiety	After the intervention, there was a trend of better physical health indicators in the Tai Chi with music group than Tai Chi only group or music only group. However, this was not significant.	Liu et al (2020) [19]	
		Coping style	There was a trend of better positive coping and negative coping in the Tai Chi with music group compared to Tai Chi only group or music only group. However, this was not significant.	Liu et al (2020) [19]	
		Inspiration and arouse	More participants in the Tai Chi with music group rated the class as effective or very effective for inspiration and arousal.	Zhao and Wang (2017) [45]	
		Fear of falling	Post fear of falling in the Tai Chi with music group was better than in the Tai Chi only group, though not statistically different.	Du et al (2017) [31]	
**Experience of Tai Chi practice**					
	**Tai Chi practice**				
		Tai Chi learning motivation	After the intervention, significantly higher motivation and concentration were found in the Tai Chi with music group compared to the Tai Chi only group.	Li (2013) [41]	
		Interest in Tai Chi	Participants self-reported interests in Tai Chi increased significantly from baseline to end of study only in the Tai Chi with music group.	Zhao and Wang (2017) [45]	
		Concentration	Better concentration was found in the intervention group; concentration was higher postintervention in the intervention group.	Li (2013) [41]	
		Enjoyment of Tai Chi practice	Beginner experienced higher enjoyment from practicing Tai Chi with all 3 types of music (more obvious effects to less: soft and relaxing, fast and joyful, and sad music) compared to more advanced, but no differences in enjoyment between beginners and more advanced were found in the Tai Chi without music group. Listening to sad music has the lowest response in both beginner and advanced groups.	Lv et al (2019) [42]	
		Perceived difficulties of learning Tai Chi	More participants rated learning Tai Chi as very difficult in the control group, while significantly more participants in the intervention group rated learning Tai Chi as easy after the intervention.	Zhao and Wang (2017) [45]	
		Self-efficacy (confidence and perceived mastering in Tai Chi practice)	More participants were confident in learning Tai Chi in the Tai Chi with music group, and more participants reported mastering Tai Chi after the intervention.	Zhang (2009) [44]; Zhao and Wang (2017) [45]	
		Tai Chi performance score^b^	Significantly better performance was found in the Tai Chi with music group.	Zhang (1999) [43]; Li (2013) [41]; Zhang (2009) [44]; Zhao and Wang (2017) [45]	
		Tai Chi class compliance	The compliance rate in the Tai Chi with music group (84%) is higher compared to the Tai Chi without music group class (71%), but the difference was not statistically significant.	Du et al (2017) [31]	
		Practice outside of class	Significantly more participants in the Tai Chi with music group practiced outside of class than in the Tai Chi only group.	Zhang (2009) [44]	
	**Perceived instruction quality**				
		Satisfaction with instruction	More participants experienced significantly better satisfaction with the instruction given in the intervention group.	Li (2013) [41]; Zhang (2009) [44]	
		Instruction quality	More participants rated very high quality of the instructions in terms of content sequency and structure, the arrangement of exercise intensity and frequency, instruction style, and overall instruction quality.	Zhao and Wang (2017) [45]	

^a^DGI: dynamic gait index.

^b^Tai Chi performance score was rated based on the Tai Chi movements in correctness, consistency, pace, and style; obvious errors, moderate errors, and minor errors were also assessed.

## Discussion

### Principal Findings

This scoping review aimed to map out the application of music in Tai Chi practice. We identified 7 studies published in either Chinese or English using controlled trials to compare the differences between practicing Tai Chi with and without music on various outcome measures. Most studies were conducted in China, and the selection and use of music for Tai Chi practice were heterogeneous. There is clear positive evidence supporting the beneficial effects of applying music to Tai Chi practice to improve the perceived quality of Tai Chi instructions and promote Tai Chi learning experience, enjoyment, concentration, adherence, and movement performance. However, further investigations are needed regarding how music should be selected in Western culture, its benefits in beginners versus advanced practitioners, and whether listening to music alone would lead to similar health benefits compared to practicing Tai Chi with music.

The studies included in this review used various types of music for Tai Chi practice, and music was used either throughout the instruction and routine practice or only for routine practice but not during instruction. In general, the music selected was soothing, soft, and relaxing [31,44,45]; aligned well with the soft, gentle, slow, and graceful movements of Tai Chi; and suitable to participant culture [31,45]. The finding is consistent with another study reviewing the psychophysical effects of music in sports and exercise [46]. The review indicated that the keys to selecting music should include considering not only the sociocultural background of the practitioners but also the nature of the physical activity and coordination with the physical activity task [46]. Therefore, music was often used in sports for pre-event preparations, warm-ups, and training sessions. In this review, music was played either throughout the Tai Chi class session or only during practicing the Tai Chi routines, both of which formats showed favorable effects [31,45]. However, since most of the included studies were conducted in China, further investigation is needed to determine the generalizability of these findings to other regions. In addition, Tai Chi has been strongly recommended to improve balance and promote health in the aging populations; while most included studies focused on Chinese college students, studies assessing to what extent the findings may apply to older populations are warranted.

Evidence from many studies suggests that music could promote positive effects during exercise, such as increasing motivation, happiness, confidence, relaxation, and performance levels [47,48]. Similarly, we found that practicing Tai Chi with music could yield higher learning motivation [41], concentration [41], enjoyment [42], confidence [43,45], performance and compliance [31,41,43-45], and lower perceived difficulties in learning Tai Chi [45]. Previous studies reported that many people who do not habitually engage in exercises often find it difficult to initiate an exercise routine, and some well-documented barriers to regular exercise engagement include a lack of motivation and enjoyment and feeling bored and physically uncomfortable [49]. The findings from the included studies may suggest that adding music to Tai Chi practice could help alleviate the barriers to engaging in Tai Chi exercise, especially for beginners. For example, among the included studies, one study found that Tai Chi beginners had a stronger positive influence from music than advanced practitioners assessed by galvanic skin responses (a signal for capturing the autonomic nerve responses) and subjective enjoyment [50]; this may indicate that Tai Chi beginners may benefit more from using music when practicing Tai Chi in comparison to more advanced users [42]. Music could likely help beginners feel relaxed and focused, and promote Tai Chi exercise in those who are new to learning Tai Chi. For more advanced Tai Chi practitioners, this may vary depending on various factors such as the practitioner’s mood and environment [36]. As an exercise that originated in Asian culture, adding music affiliated with Western populations may help promote its instruction, implementation, and dissemination. However, further investigations are needed to confirm this assumption.

The findings differ when assessing improvements in health outcomes through adding music. Balance, as measured by the dynamic gait index, was found to be significantly improved in the study participants incorporating music, but not in their counterparts practicing Tai Chi without music after a 15-week Tai Chi intervention. Balance is a frequently studied measurement of Tai Chi’s effects on health [51], and the Centers for Disease Control and Prevention also highly recommends that older adults practice Tai Chi to improve balance [52]. For other assessed health measurements, although not significant, trends showed better outcomes in the Tai Chi with music group such as physical symptoms and depression. For other health measurements, no significant improvements were detected, which could be due to the relatively short intervention lengths. Further research with longer interventions and a larger sample size is warranted to explore the possible symbiotic effect of Tai Chi and music on health outcomes.

**Figure 2:**
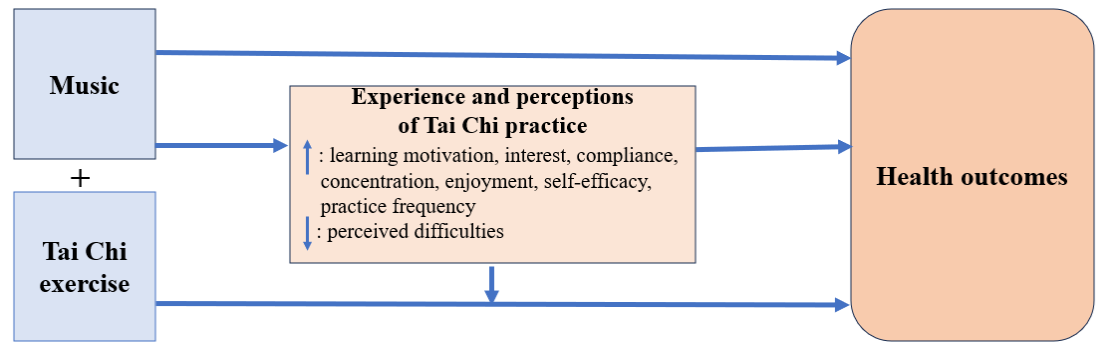
Conceptual framework of applying music to Tai Chi practice to increase Tai Chi’s health benefits.

Integrating music into Tai Chi practice may offer a promising avenue for enhancing its benefits on health outcomes in a holistic approach. First, based on the findings of this review, the health benefits could be through the pathway of a positive influence of added music on perceived enjoyment and concentration, improved adherence or compliance, and overall positive experience. In addition, as evidenced in the literature, listening to music alone could improve a variety of health outcomes including but not limited to mental health, cognitive function, and cardiovascular health [53-58]. Therefore, we hypothesized a theoretical framework of adding music to Tai Chi practice to increase its benefits on health outcomes (Figure 2). Future research could focus on whether the application of music to Tai Chi practice leads to synergistic effects on health outcomes. This includes both physical (eg, mobility, balance, and metabolic health), cognitive (eg, orientation and memory), and mental health (eg, stress and depression symptoms). Studies could also aim to quantify these outcomes and understand the underlying mechanisms driving these potential benefits. It is also warranted to investigate which music (such as type of music, its tempo, and rhythm) is appropriate to maximize health benefits, and how these factors might influence different populations such as various age groups and health conditions. These investigations may provide insights into how to improve health through a holistic approach. Last, but not least, qualitative studies to gather in-depth insights from participants on their experiences, preferences, and perceptions of Tai Chi practice with music may provide valuable information to guide the development of tailored and effective Tai Chi exercise programs. Tools and recommendations could be developed to guide interventions using music to Tai Chi practice for health benefits.

### Limitations

The findings of this scoping review should be interpreted with caution due to the majority of included studies being conducted in China among college students. Even though music has been used during Tai Chi practice in other populations as reported in prior studies [26], the mechanisms of using music in Tai Chi practice in various populations have been understudied. Further research is needed to explore the mechanisms and the potential harms or benefits and establish the practicality of using music for Tai Chi practice in other regions and populations. In addition, the heterogeneous interventions, lengths, and frequencies of the included studies may further limit the application of the findings.

### Conclusions

Based on the findings of the included studies, Tai Chi style or form selection, the music used during Tai Chi practice, and the studied populations were heterogeneous. Various outcomes, such as differences in Tai Chi practice experience, instruction quality, and health outcomes, were assessed. There is positive evidence supporting the beneficial effects of applying music to Tai Chi practice to improve the perceived quality of Tai Chi instructions and promote Tai Chi learning experience, enjoyment, concentration, adherence, and movement performance. Particularly, including music in Tai Chi practice may promote Tai Chi among individuals new to Tai Chi practice through perceived better instruction and learning experience. Therefore, applying music to Tai Chi learning and practice might help promote the implementation of Tai Chi in the general population. However, further study with a rigorous study design is needed to clarify if there are synergistic effects of Tai Chi and music on health outcomes.

## Appendix

Multimedia Appendix 1 PRISMA-ScR (Preferred Reporting Items for Systematic reviews and Meta-Analyses extension for Scoping Reviews) checklist.

Multimedia Appendix 2 Search strategies used on PubMed for finding research articles about the application of music in Tai Chi exercise.

## Abbreviations

PRISMA-ScR: Preferred Reporting Items for Systematic Reviews and Meta-analysis extension for Scoping Reviews
