# Supplementary material for: Current Evidence of the Application of Music in Tai Chi Exercise: Scoping Review
Source: Asian Pac Isl Nurs J. 2024 Sep 19;8:e60104. doi: 10.2196/60104 (PMC11450349; doi:10.2196/60104)
Supplement: Multimedia Appendix 2 [file apinj_v8i1e60104_app2.docx]

**PubMed**

| Search | Term/terms | Number of articles |
| --- | --- | --- |
| S1 | Tai Chi Chuan [MESH] | 1,402 |
| S2 | Tai-ji [Title/Abstract] | 64 |
| S3 | **(Tai Chi [Title/Abstract])** | 2,116 |
| S4 | **(Chi, Tai [Title/Abstract])** | 7 |
| S5 | (Tai Ji Quan [Title/Abstract]) | 34 |
| S6 | (Ji Quan, Tai [Title/Abstract]) | 1 |
| S7 | (Quan, Tai Ji [Title/Abstract] | 2 |
| S8 | (Taiji [Title/Abstract]) | 119 |
| S9 | (Taijiquan [Title/Abstract]) | 51 |
| S10 | (T'ai Chi [Title/Abstract]) | 121 |
| S11 | (Tai Chi Chuan [Title/Abstract])) | 216 |
| S12 | S1 OR S2 OR S3 OR S4 OR S5 OR S6 OR S7 OR S8 OR S9 OR S10 OR S11 | 2,494 |
| S13 | (Music [Title/Abstract]) | 20,655 |
| S14 | S12 AND S13 | 46 |
